# The Emergence of Mania After Initiating Escitalopram for Anxiety Disorder

**DOI:** 10.7759/cureus.63441

**Published:** 2024-06-29

**Authors:** Kamalakar Surineni, Austin Armstrong, Sara Wallace, Nolan Schrader

**Affiliations:** 1 Psychiatry and Behavioral Sciences, University of Kansas School of Medicine Wichita, Wichita, USA

**Keywords:** affective switch, generalized anxiety disorder (gad), bipolar switch, escitalopram, bipolar disorder

## Abstract

The abrupt transition into mania, known as a bipolar switch, poses a significant challenge in the treatment of mental illnesses. We present a case of a 22-year-old Hispanic female with generalized anxiety disorder (GAD) and autism spectrum disorder (ASD) who developed mania within five days after initiating escitalopram 5 mg. The patient had no reported history of bipolar disorder prior to this episode, and an extensive medical workup ruled out organic causes. The patient was in the acute inpatient psychiatric unit for 25 days and returned to baseline after discontinuation of escitalopram and initiating divalproex and olanzapine. This case underscores the potential risk of a bipolar switch with antidepressant use and highlights the importance of vigilant monitoring and considering underlying bipolarity in such patients.

## Introduction

Bipolar disorders (BD) present a unique set of diagnostic challenges to clinicians as their initial presentation is often unipolar depression [1]. Delineating between these diagnoses is further complicated if the mania is not severe enough to prompt the patient to seek medical attention or if essential parts of the diagnostic interview are difficult to obtain. Co-occurring substance abuse further complicates the diagnosis. The bipolar switch might arise due to spontaneous or iatrogenic causes [2]; a variety of medications have been implicated in “treatment-emergent affective switch,” including all major antidepressant classes [3], stimulants [4], corticosteroids [5], and thyroid hormones [6]. Bipolar patients not on a mood stabilizer are particularly susceptible to treatment-emergent affective switch. The implications of a bipolar switch can be severe, necessitating heightened clinical awareness and proactive management strategies [7-9]. Additionally, clinicians may be more hesitant to prescribe mood-stabilizing medications, especially for first episodes [10], and more readily attribute symptoms to other psychiatric conditions or substance use [11]. Past studies have demonstrated worse long-term outcomes and accelerated mood cycling among bipolar patients who experience antidepressant-induced bipolar switch [12]. Accordingly, clinicians of all disciplines must remain aware of potential inciting events and at-risk populations for treatment-emergent mania.

Selective serotonin reuptake inhibitors (SSRIs) are well-established precipitants of bipolar switching, with estimated rates ranging between 3 and 6% in BD and less than 3% in major depressive disorder [12]. Escitalopram may be more prone to inducing this phenomenon due to its high selectivity for serotonin transporter (5-HTT) [13]. Escitalopram is a popular choice for the treatment of depression and anxiety due to its high efficacy, limited side-effect profile, and low risk for drug interactions [13]. Although there are several reports of escitalopram-induced mania, most of these cases occur at doses at or exceeding 10 mg. This is consistent with previous reports suggesting a dose-dependent relationship between treatment-emergent switching and escitalopram use [12,14]. The precipitation of mania with low-dose (5 mg) escitalopram in the present case suggests a need for consistent monitoring even at low doses.

It is important that clinicians are aware of patient populations susceptible to treatment-emergent-affective switching before the initiation of pharmacotherapy with known triggers. The present case discusses the precipitation of mania in a treatment-naïve female with comorbid diagnoses of generalized anxiety disorder (GAD) and autism spectrum disorder (ASD). Although both GAD and ASD are well-established comorbid conditions of bipolar disorder (BD) [15,16], their influence on risk as it relates to treatment-emergent affective switching is not well understood.

## Case presentation

Ms. L is a 22-year-old Hispanic female who was seen for a new patient intake after a referral from her psychologist. She had past diagnoses of generalized anxiety disorder (GAD) and autism spectrum disorder (ASD)-high functioning. She had never been on any psychiatric medications, and there were no known mental illnesses in her family. This patient had been engaged in psychotherapy for the previous 18 months for the treatment of anxiety with mild improvement. 

During the initial evaluation, Ms. L was anxious and reported excessive worry about various things in general. She was diagnosed with GAD, social anxiety disorder, and ASD and was started on escitalopram 5 mg to treat symptoms related to anxiety. After taking escitalopram for five days, the patient became irritable, disorganized, and confused and was brought to the emergency department for evaluation. 

**Figure 1:**
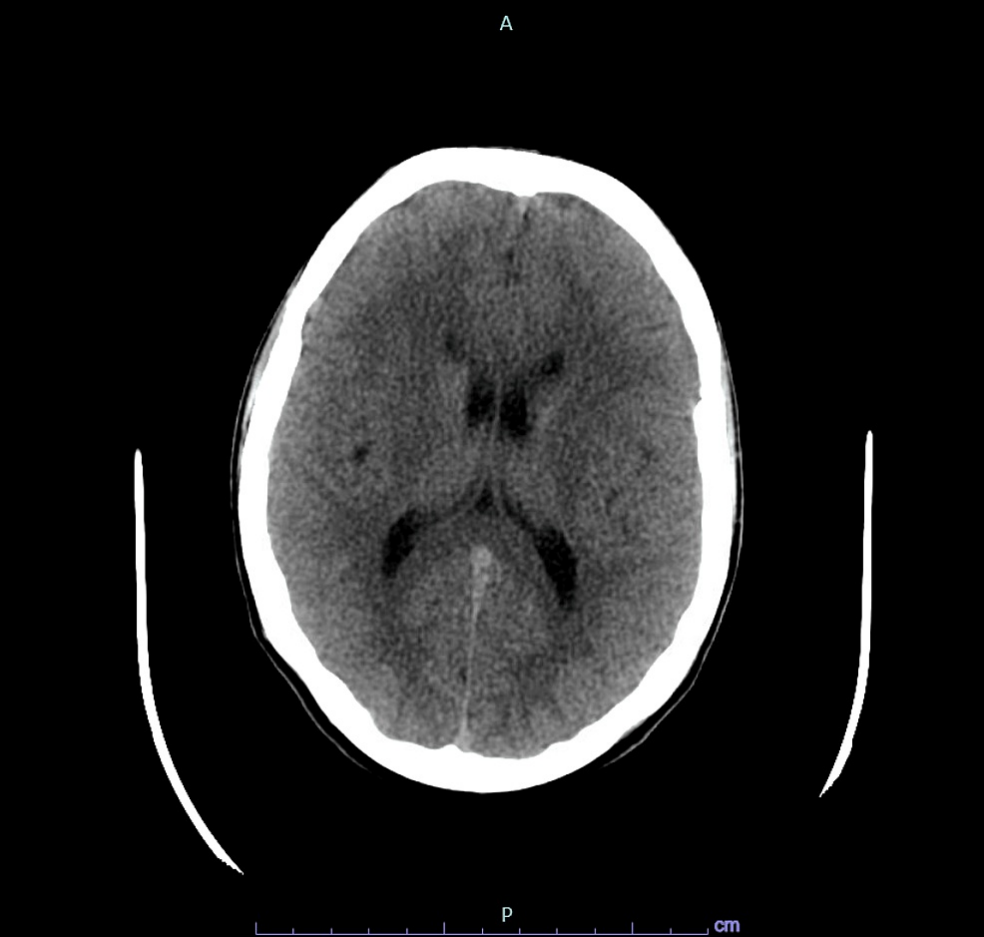
CT head without contrast showing no abnormalities CT= Computed Tomography

Upon evaluation in the emergency department, Ms. L was found to be minimally cooperative, disorganized in her thought process, disheveled with poor eye contact, irritable, and inattentive. She demonstrated increased volume and quantity of speech. All aspects of her medical workup, including complete blood and metabolic counts, urine drug screen, and head CT without contrast, were within normal limits (Figure 1).

There was concern for autoimmune encephalopathy, and a lumbar puncture was performed. Cerebrospinal fluid (CSF) was negative for infection, the CSF chemistry panel was unremarkable, the paraneoplastic and infectious panel was negative, and the autoimmune panel was negative. Both blood and CSF cultures were ordered, and the results were negative (Table 1).

**Table 1:** CSF analysis showing no abnormalities ruling out autoimmune causes for patient's presentation CSF: Cerebrospinal fluid, N.A.: Not Applicable, Ab: Antibody,CBA: Cell-Based Assay, NMDA-RAD: N-Methyl-D-Aspartate Receptor Antibody Disease, VDRL : Venereal disease research laboratory, GAD65 Ab: Glutamic Acid Decarboxylase 65 Antibody, W Nile Igg: West Nile Virus Immunoglobulin G, W Nile IgM: West Nile Virus Immunoglobulin M, DPPX AD IFA: Dipeptidyl-Peptidase-Like Protein 6 Autoimmune Encephalitis Indirect Fluorescent Antibody test,GFAP IFA: Glial Fibrillary Acidic Protein Indirect Fluorescent Antibody test, mGluR1 Ab IFA: Metabotropic Glutamate Receptor 1 Antibody Indirect Fluorescent Antibody test, ANNA1: Anti-neuronal Nuclear Antibody Type 1 in CSF, ANNA2: Anti-neuronal Nuclear Antibody Type 2 in CSF, ANNAS: Anti-neuronal Nuclear Antibodies, AGNA1: Anti-Glial/Nuclear Autoantibodies Type 1 in CSF, PCA-1 Ab: Purkinje Cell Cytoplasmic Autoantibody Type 1 in CSF, PCA2: Purkinje Cell Cytoplasmic Autoantibody Type 2 in CSF, PCATR: Purkinje Cell Cytoplasmic Antibody, Type Tr, Amphip Ab: Amphiphysin antibody in CSF, CRMP-5-IgG: Collapsin Response Mediator Protein 5 Immunoglobulin G in CSF, AMPA-R ab: AMPA Receptor antibody in CSF, GABA-B-R Ab: GABA-B Receptor antibody in CSF, LGi1-IgG CBA-CSF: leucine‐rich glioma‐inactivated 1

Test Name	Result	Ref Range
Glucose CSF	65 mg/dL	60 to 70 mg/dl
Protein CSF	34 mg/dL	15 to 45 mg/dl
IgM Immunoglobulin CSF	0 mg/dL	0.0-0.7 mg/dL
IgG Immunoglobulin CSF	2.7 mg/dL	0.0-6.0 mg/dL
IgA Immunoglobulin CSF	0.2 mg/dL	0.0-0.7 mg/dL
Helicobacter IGG	0.18 mg/dL	<8.95 mg/dL
C-Reactive Protein	<0.1 mg/dL	<0.5 mg/DL
NMDA-RAD CBA CSF	Negative	N.A.
VDRL Screen CSF	Negative	N.A.
GAD65 Ab CSF	Negative	N.A.
W Nile Igg CSF	Negative	N.A.
W Nile IgM CSF	Negative	N.A.
DPPX AD IFA, CSF	Negative	N.A.
GFAP IFA, CSF	Negative	N.A.
mGluR1 Ab IFA, CSF	Negative	N.A.
ANNA1 CSF	Negative	N.A.
ANNA2 CSF	Negative	N.A.
ANNAS CSF	Negative	N.A.
AGNA1 CSF	Negative	N.A.
PCA-1 Ab CSF	Negative	N.A.
PCA2 CSF	Negative	N.A.
PCATR CSF	Negative	N.A.
Amphip Ab CSF	Negative	N.A.
CRMP-5-IgG CSF	Negative	N.A.
Reflex Added	None	N.A.
AMPA-R ab CSF	Negative	N.A.
GABA-B-R Ab CSF	Negative	N.A.
LGl1-IgG CBA-CSF	Negative	N.A.

In addition to disorganized behavior, disorganized thought processes, and increased volume, quantity, and rate of speech, Ms. L exhibited a decreased need for sleep (less than three hours of sleep), psychomotor agitation, euphoric mood, and racing thoughts. Escitalopram was discontinued, and the patient was started on lorazepam (1 mg at bedtime for sleep), olanzapine (2.5 mg and titrated to 12.5 mg), and divalproex (500 mg at bedtime and titrated to 750 mg). 

She required frequent PRN (pro re neta) medications for behaviors in the first week of hospitalization, though this decreased as the patient’s sleep improved and the medication regimen was advanced. The patient was discharged on day 25 and instructed to follow up with outpatient psychiatry, where she was tapered off olanzapine and lorazepam and maintained on divalproex.

## Discussion

The risk of bipolar switch with antidepressant initiation in treatment-naïve patients is a critical consideration, as illustrated by the present case and supported by earlier reports [2,3,12]. Most instances of bipolar switch occur within two to three weeks of medication initiation or dose adjustment, necessitating vigilant monitoring, particularly during these periods [12]. Observational studies also found some risk factors which could help predict bipolar switch, including hypersomnia, psychomotor retardation, family history of mania, earlier age of onset, greater number of episodes, greater proportion of time ill, postpartum episodes, and/or poor response to antidepressants [17]. In addition, literature suggests anxiety disorders and ASD are highly comorbid with BD [16]. It is postulated by previous studies that there is a genetic link between BD and anxiety disorders. Children whose parents have bipolar disorders experience a higher risk for anxiety, perhaps due to serotonin transporter genotypes conferring a higher risk for BD [17]. In the present case, these comorbid conditions may have lowered the threshold for a manic episode such that low-dose escitalopram was sufficient to induce mania. This case accentuates the need to obtain a thorough patient history, including any prior hypomanic or manic symptoms, as well as family psychiatric history to aid in identifying individuals at heightened risk.

Screening for risk factors may also include a genetic component. In one study, the *SLC6A4* (serotonin transporter-linked polymorphic region (5-HTTLPR)) serotonin transporter gene showed two polymorphisms that were associated with different response rates to SSRI treatment in Bipolar I. The S allele was not associated with clinical risk of bipolar switch; however, the L-A-10 haplotype was associated with reduced risk (p=.012) [18,19]. These findings indicate a greater need for further study of the possible genetic role in predisposing a patient to SSRI-induced mania. 

While previous case reports show bipolar switch occurring in patients who did not have a pre-existing bipolar diagnosis, it is still unclear whether this is uncovering an already existing bipolar illness, or whether a secondary mania occurred with no bipolar progression. Currently, there is insufficient longitudinal data to make this delineation.

## Conclusions

We present the case of a 22-year-old female with GAD and social anxiety disorder who developed mania following the initiation of low-dose escitalopram. This case accentuates the need for thorough history and screening to identify patients at risk for treatment-emergent mania. The consequences of failing to recognize and respond to bipolar switch are severe and include prolonged hospitalization. As demonstrated by the present case, patients with comorbid conditions related to the development of BD, like anxiety disorders and ASD may be particularly susceptible to bipolar switching and should be monitored closely during the initiation and titration of treatment. Clinicians should also be aware of the patient’s family history due to the potential hereditary nature of bipolar disorder, as well as specific genetic polymorphisms thought to confer an increased risk of treatment-emergent switching.
